# A Case Report of an Upper Cervical Spine Osteolipoma With Unusual Clinical Presentation

**DOI:** 10.7759/cureus.109135

**Published:** 2026-05-18

**Authors:** Omer Alkhateeb, Michael Spiess

**Affiliations:** 1 Orthopedic Surgery, University of Saskatchewan, Saskatoon, CAN; 2 Spine Surgery, University of Saskatchewan, Saskatoon, CAN

**Keywords:** cervical spine tumor, ossifying lipoma, osteolipoma, spine cord, upper cervical spine

## Abstract

Osteolipoma is an uncommon benign lipomatous tumor characterized by the presence of mature adipose tissue intermixed with lamellar bone. We present a unique case of a 22-year-old female with an osteolipoma in the upper cervical paraspinal region, closely associated with the vertebral artery. The patient exhibited atypical symptoms, including intermittent dizziness and numbness around the right eye, which were previously unreported in similar spinal cases. Diagnosis was confirmed through multimodal imaging and CT-guided biopsy. Surgical excision was performed without complications, and the patient experienced complete symptom resolution at follow-up. This report contributes novel clinical and anatomical insights to the limited literature on spinal osteolipomas, emphasizing the importance of individualized evaluation and multidisciplinary management for optimal outcomes.

## Introduction

Osteolipoma is considered a lipoma with a mesenchymal element [1-4]. It can be defined as having mature adipose tissue intermixed with lamellar bone [5]. Osteolipoma is a rare variant of the benign lipoma tumor, accounting for less than 1% of lipomatous lesions [3,4]. However, there are diverse mesenchymal components that can present within a lipoma, including blood vessels (angiolipoma), fibrous connective tissue (fibrolipoma), cartilaginous metaplasia (chondrolipoma), and osseous metaplasia (osteolipoma) [5]. It is crucial to distinguish that osteolipoma, also referred to as "ossifying lipoma," differs from interosseous or periosteal lipomas, in which a lipoma undergoes independent ossification without a bony connection [1-5].

The existing literature predominantly reports osteolipomas occurring in the head and anterior neck region, as well as the buccal mucosa. Fewer cases have been documented in other areas of the body, with only ten cases identified in proximity to the posterior spine [1-4,6-13]. Importantly, our case was the only one in very close proximity to the vertebral artery. In addition, it had different symptoms (e.g., episodes of dizziness, intermittent numbness around the eye), which we did not observe with the other reported spinal cases in the literature so far.

## Case presentation

A 22-year-old female patient was referred to the Orthopedic Spine outpatient clinic for further evaluation and management of her slowly growing palpable posterior neck mass. The mass was first noticed in the right paraspinal upper neck region during a massage session in the spring of 2023. This was further investigated by her chiropractor with an ultrasound, which was negative. However, she was also seeing her physiotherapist for exercises to relieve her pain. Her physiotherapist ordered another ultrasound, which suggested a posterior neck calcified mass. Thereafter, the patient was referred to the spine clinic, and further investigation, such as MRI, CT scan, PET scan, and CT-guided biopsy, was requested. On further assessment in our clinic, it seemed that the mass was non-bothersome and asymptomatic initially. However, the patient reported a history of intermittent headaches for three years prior, which had gradually worsened over time.

The patient described the headaches as a band-like pain, associated with episodes of dizziness, nausea, and intermittent numbness around the right eye. These neurological symptoms were exacerbated by sleeping in the supine position, lifting heavy objects, overhead reaching, neck hyperextension, and computer work. The headaches were alleviated with over-the-counter pain medication, such as acetaminophen and naproxen, as well as by sleeping in the prone position. In addition, her headaches improved with massage sessions. Unfortunately, the patient's symptoms progressively worsened after the identification of the neck mass, to the extent that it began to affect her work performance, necessitating increased analgesic use and limiting her ability to exercise. There were no associated symptoms of photophobia, phonophobia, balance or coordination difficulties, or neurogenic bowel/bladder dysfunction. Additionally, the patient did not report any pain, numbness, weakness, or paresthesias radiating to the upper or lower extremities.

The patient's medical history was significant for asthma, right wrist tendinitis, benign joint hypermobility, and postural tachycardia syndrome. Physical examination revealed a firm, tender, and non-mobile mass in the right upper cervical paraspinal region, extending from the occipital protuberance to the C2-C3 level. Neurological examination of the upper and lower extremities was within normal limits, with no signs of myelopathy.

Diagnostic imaging, including intravenous contrast CT scan, intravenous contrast MRI, PET-CT, and cervical spine radiographs, in conjunction with a CT-guided biopsy, confirmed the diagnosis of an osteolipoma. The CT scan demonstrated a large fatty mass with coarse calcifications in the right posterior neck (Figures 1-3).

**Figure 1:**
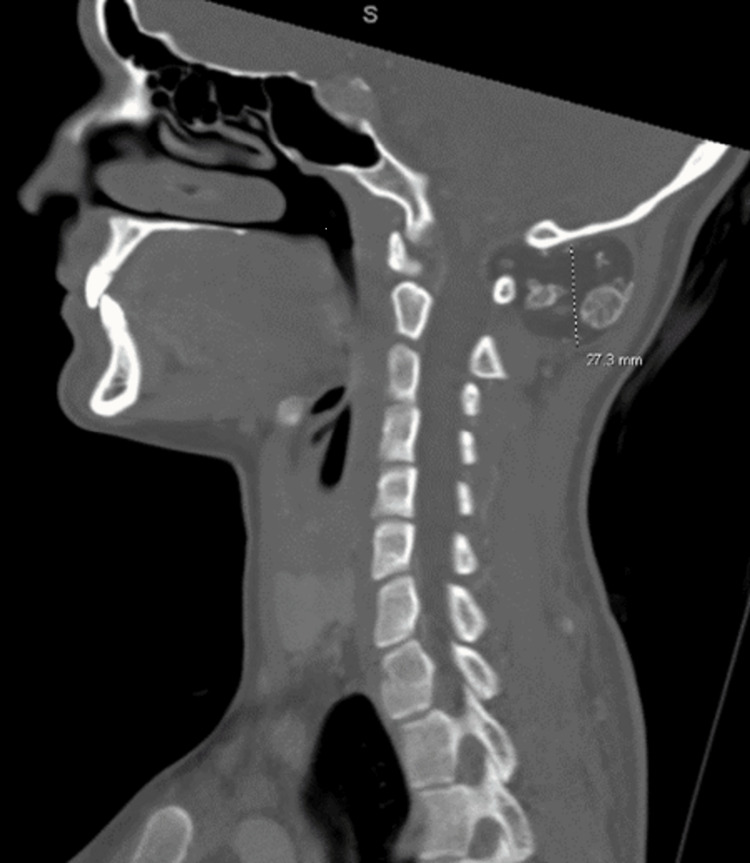
Sagittal CT scan demonstrating a large fatty mass below the occiput.

**Figure 2:**
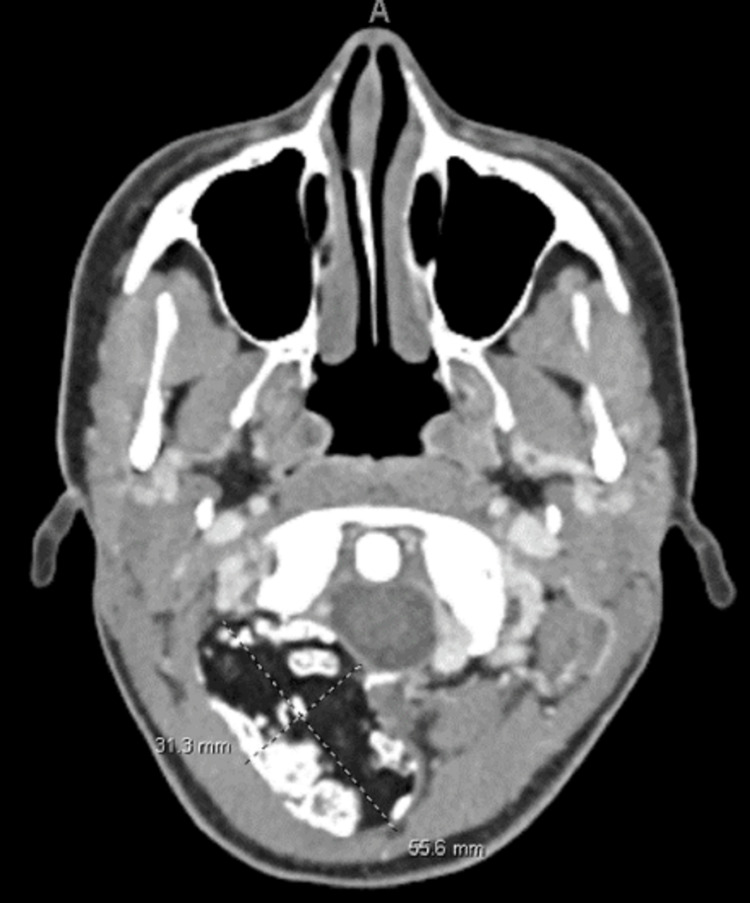
Axial CT scan soft tissue window demonstrating the same large fatty mass with coarse calcifications in the right posterior neck.

**Figure 3:**
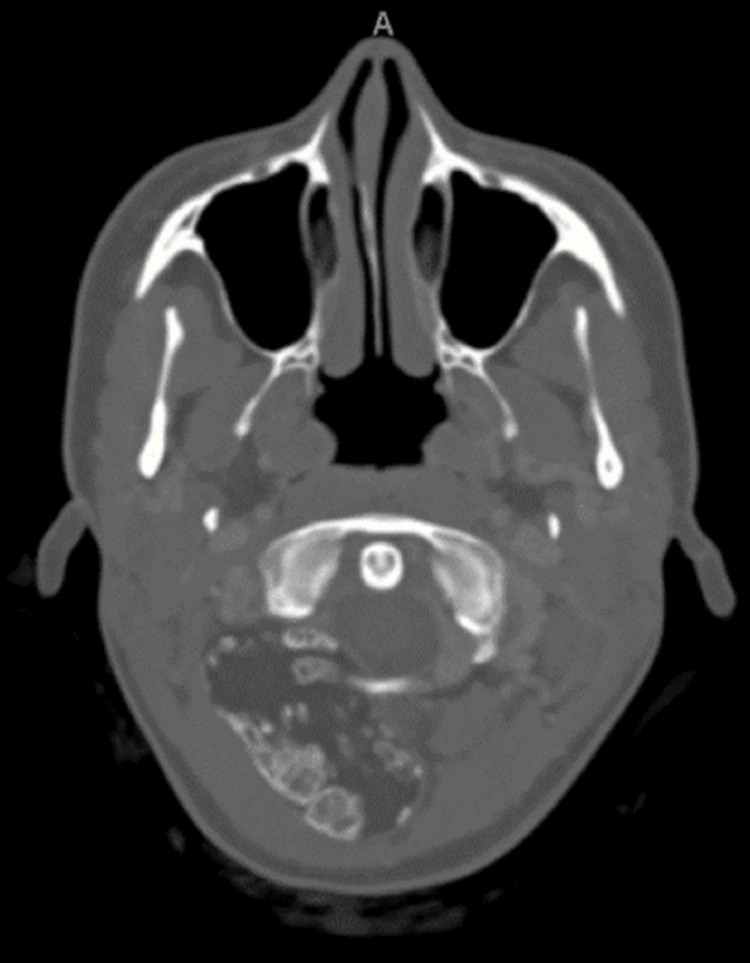
Axial CT scan bone window showing the large fatty mass in the right posterior neck between C1 and C2.

While the MRI revealed a well-circumscribed lesion in the right paraspinal region, spanning from the occiput to C2, adherent to the dorsal dura but non-compressive. The mass was also found to be in contact with the V3 segment of the right vertebral artery, without evidence of complete encasement or compression (Figures 4-7).

**Figure 4:**
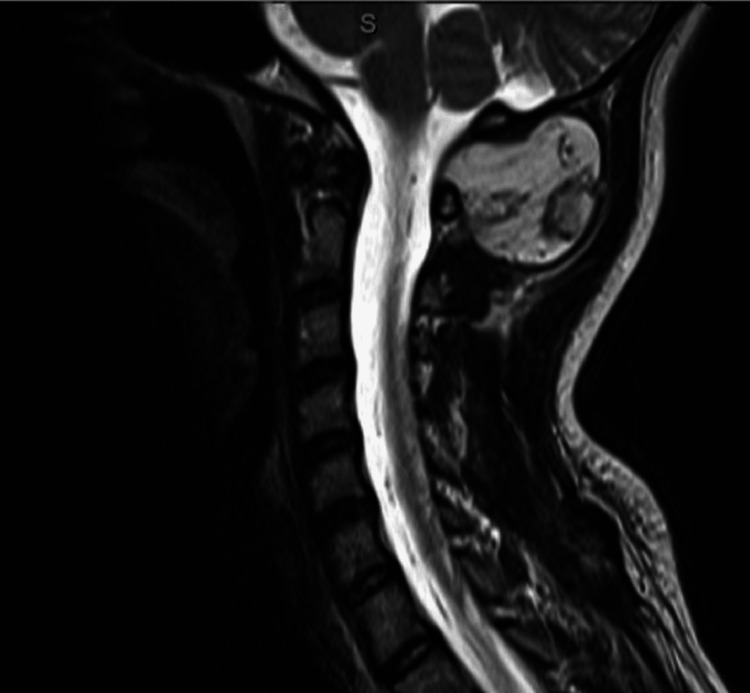
Sagittal T2-weighted cervical spine MRI showing a well-circumscribed lesion in the paraspinal region, spanning from the occiput to C2, measuring 5.3 × 3.1 × 2.8 cm, adherent to the dorsal dura but non-compressive.

**Figure 5:**
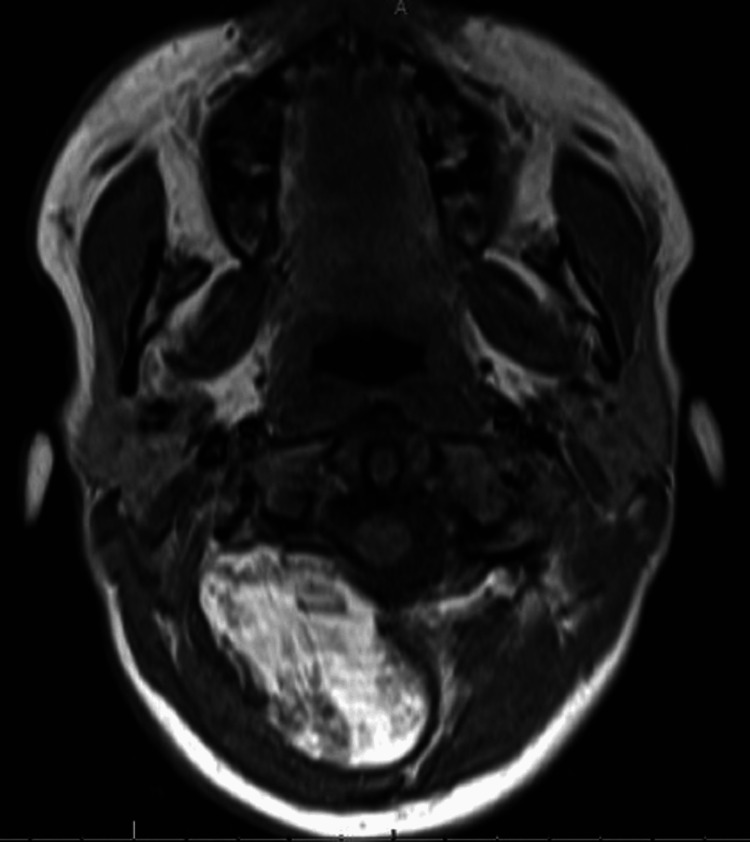
Axial T2-weighted upper cervical spine MRI showing the mass in the right paraspinal region being in contact with the V3 segment of the right vertebral artery without evidence of complete encasement or compression.

**Figure 6:**
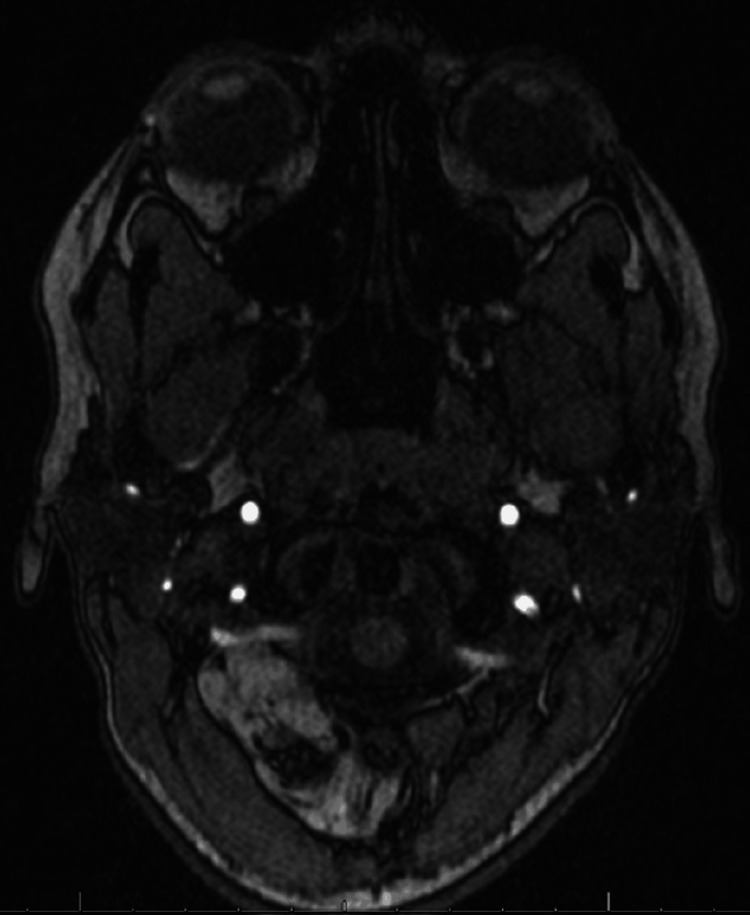
Axial T1-weighted upper cervical spine MRI with contrast showing enhancement within the mature osseous elements within the lesion, which favors enhancing marrow within mature bone.

**Figure 7:**
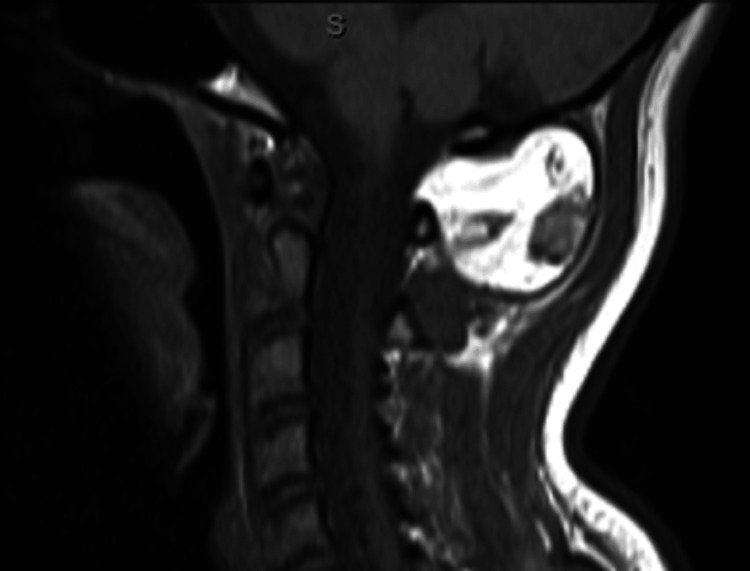
Sagittal T1-weighted MRI showing partial calcification within the mass in the paraspinal region of the cervical spine.

The PET-CT scan showed low-grade metabolic activity within the lesion. Cervical spine radiographs further corroborated the presence of subtle coarse calcifications within the mass (Figure 8).

**Figure 8:**
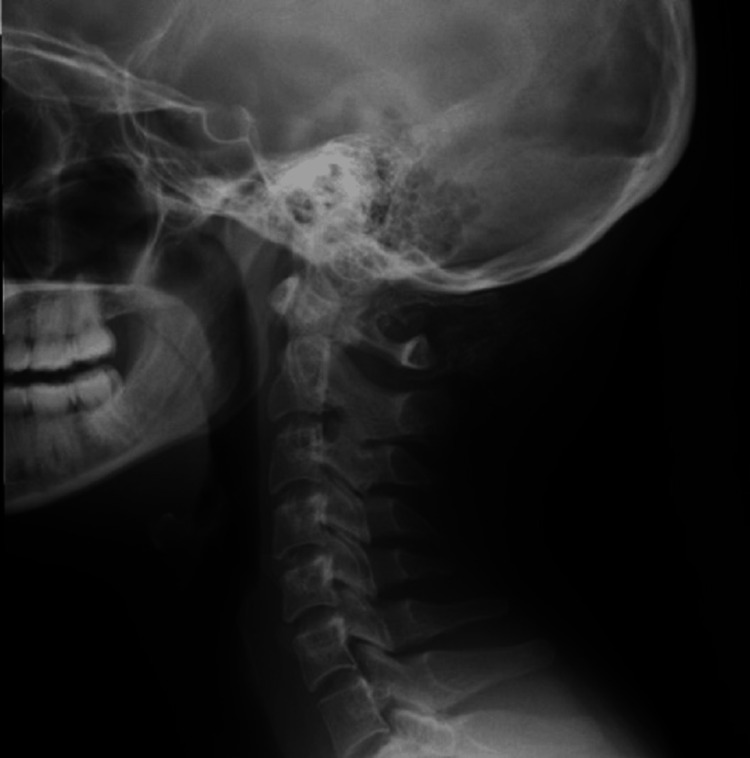
Lateral cervical spine X-ray with coarse calcification in the posterior upper neck.

The percutaneous CT-guided biopsy favored a variant of benign lipoma with some metaplastic changes, consistent with the diagnosis of osteolipoma (Figures 9-13).

**Figure 9:**
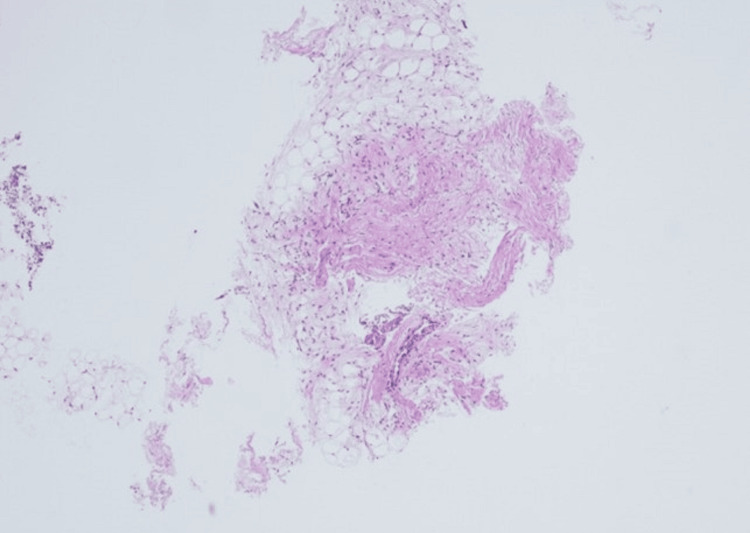
Low-power view from the CT-guided biopsy showing predominant adipose tissue with minimal fibrous stroma.

**Figure 10:**
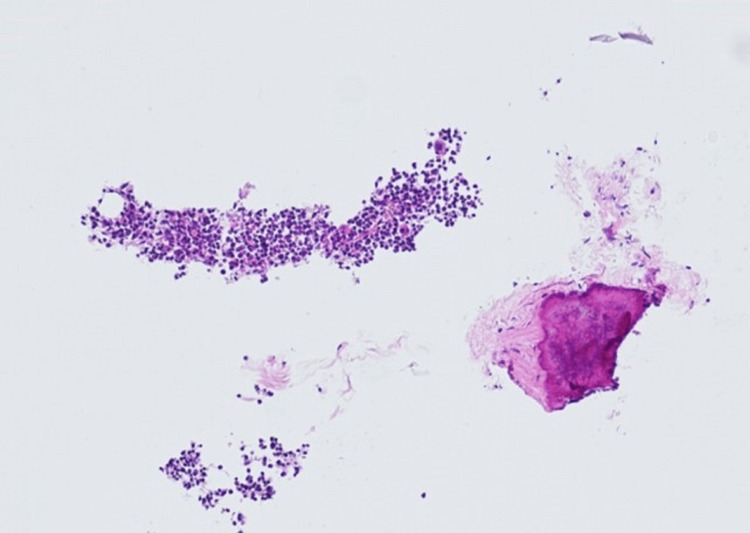
Intermediate magnification from the CT-guided biopsy revealing mixed cellular architecture with denser fibrous tissue adjacent to adipose-rich areas.

**Figure 11:**
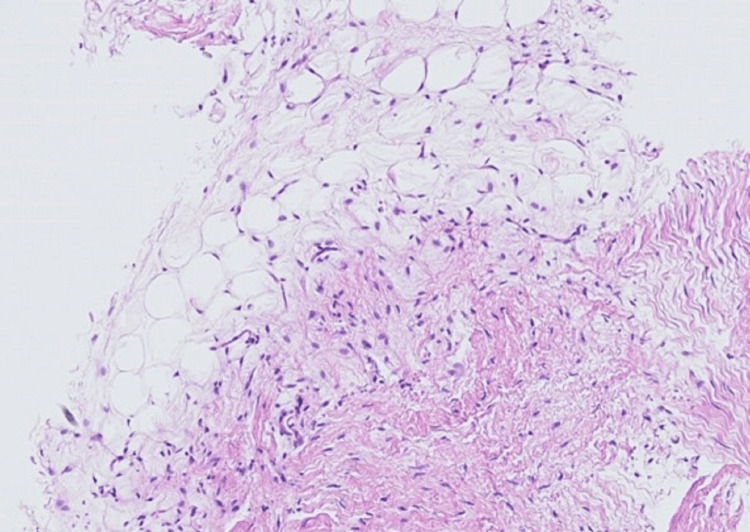
Mid-power view from the CT-guided biopsy illustrating fibrous connective tissue interdigitating with adipose lobules.

**Figure 12:**
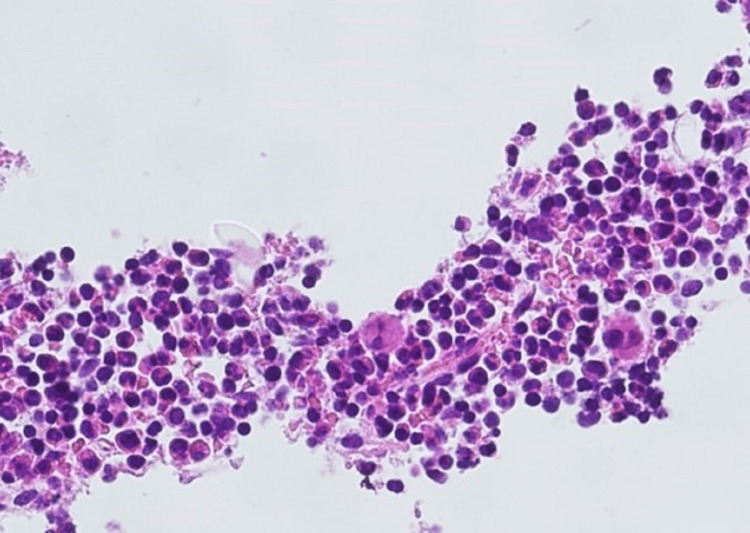
High-power view from the CT-guided biopsy showing cellular aggregates with prominent nuclei, which are consistent with marrow.

**Figure 13:**
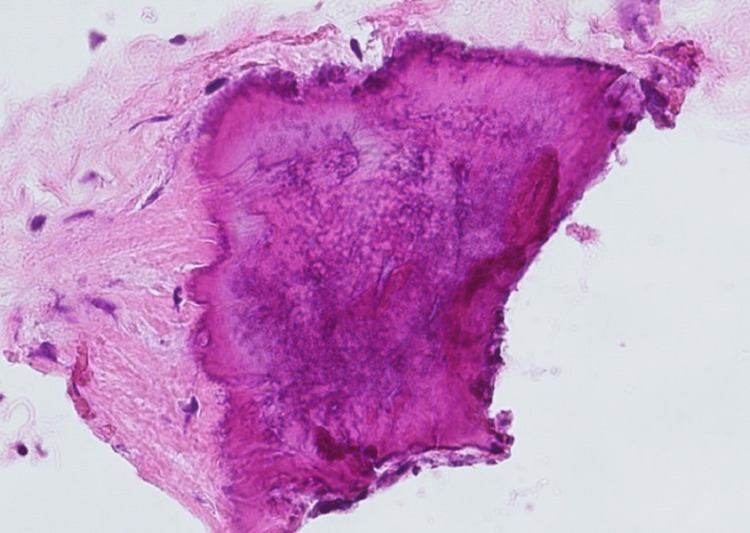
High-power view section showing central necrosis bordered by inflamed connective tissue suggestive of tissue injury.

Despite our confirmed diagnoses via a CT-guided biopsy, an urgent referral to neurology was carried out, given the intermittent numbness around the patient’s right eye and her main complaint of headaches. This was made to make sure no other treatments or investigations were needed prior to proceeding with surgical excision. Her neurologist had the same history and added that she did not have any lateralizing symptoms except for the numbness around her right eye. The neurologist’s impression was that these symptoms were nonspecific, and it is reasonable to attribute her pattern of headache to the lump in her neck, which supported the decision to excise the mass.

Intraoperative finding

Following the confirmation of the diagnosis, the patient underwent surgical excision of the osteolipoma lesion. Under general anesthesia and administration of perioperative antibiotics, the neural monitoring needles were applied to the scalp, arms, and legs, and the patient was positioned prone on an OSI table. A standard midline cervical incision was made. Dissection was carried out to expose the base of the skull, the C2 and C3 spinous processes, and the posterior aspect of the C1 ring on the left side. The firm, well-circumscribed mass was identified and carefully dissected using low-power cautery and Metzenbaum scissors, taking care to dissect around the capsule of the lesion. Despite preoperative imaging indicating proximity of the mass to the vertebral artery, intraoperative findings did not necessitate surgical exposure of the vessel, and the vascular clips and supplies prepared on the back table remained unused. Gross total excision of the mass was achieved, with no palpable residual tissue or fragments remaining (Figure 14).

**Figure 14:**
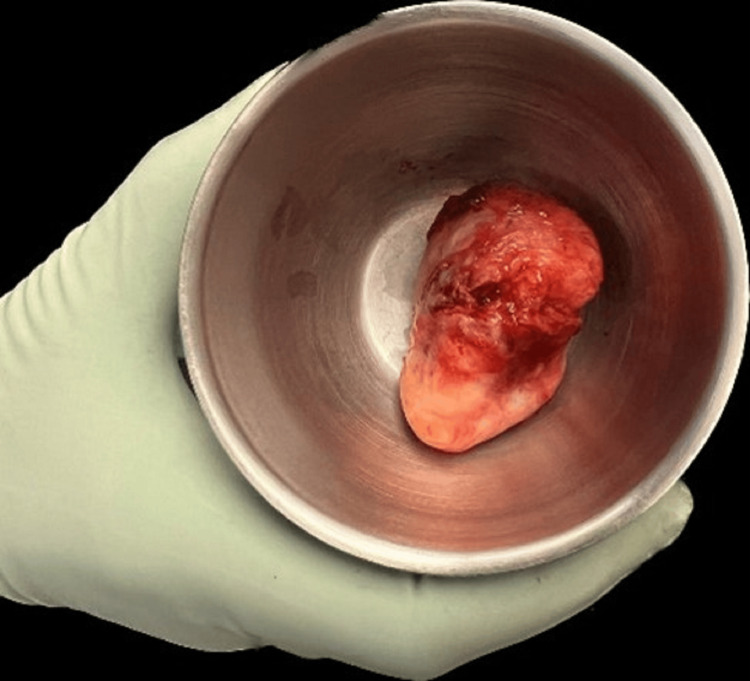
Macroscopic image of the resected lesion, intraoperatively measuring 5.5 × 3.1 × 3.6 cm, yellow to tan encapsulated rubbery to hard fibrofatty tissue with multiple hard areas of possible bone tissue extending within most of the specimen.

Intraoperatively, there was no evidence of further compression on the dorsal aspect of the spinal canal, and hemostasis was readily achieved. The surgical specimen was sent for histopathological examination. The patient tolerated the procedure well and had an uneventful postoperative course, being discharged from the hospital on the first postoperative day. The pathological mass was examined under microscopy and reconfirmed as osteolipoma (Figures 15-20).

**Figure 15:**
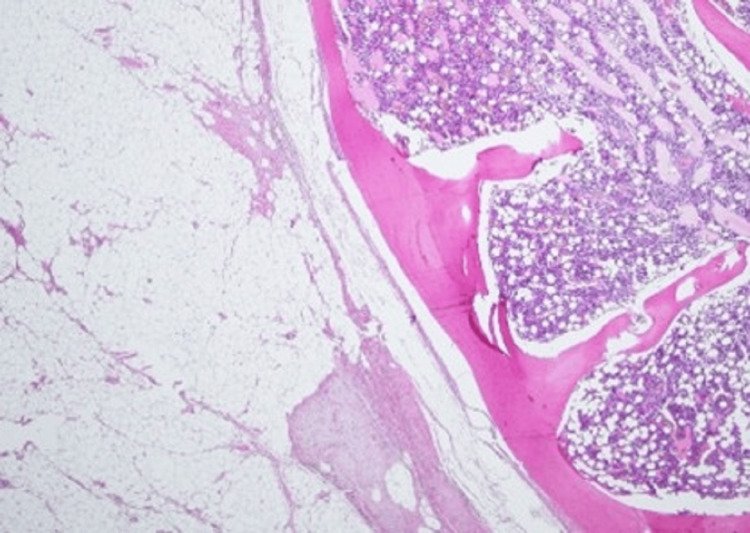
Histological section showing trabecular bone surrounded by marrow and adipose tissue consistent with osteolipoma.

**Figure 16:**
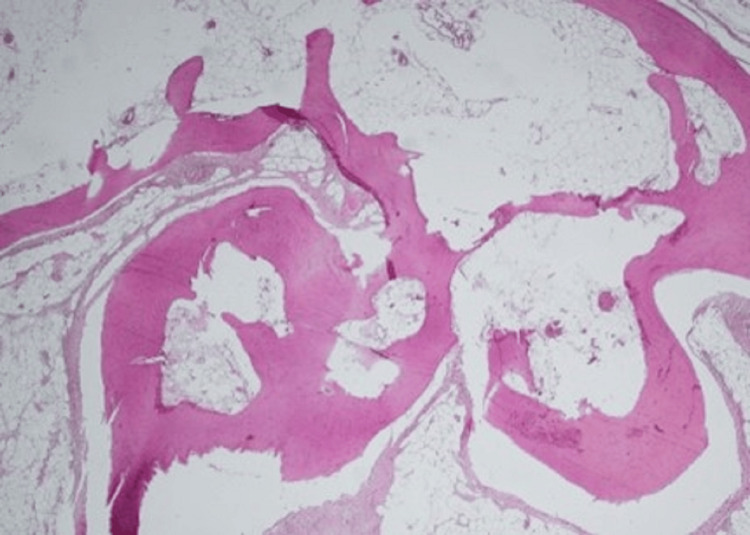
Low-power view of cancellous bone with intervening marrow spaces illustrating the osseous component of the excised lesion.

**Figure 17:**
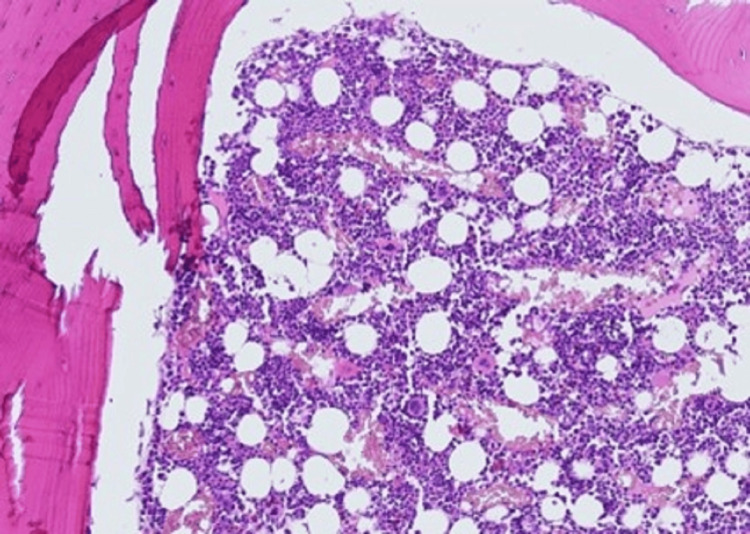
Bone marrow section showing erythroid precursors and adipocytes reflecting active hematopoiesis within the osteolipoma.

**Figure 18:**
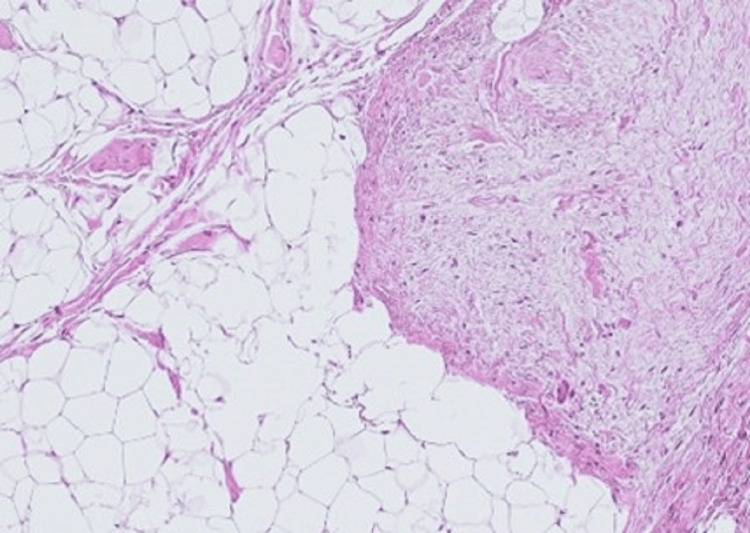
A section showing juxtaposition of the mature adipose tissue and fibrous stroma supporting the composite nature of the lesion.

**Figure 19:**
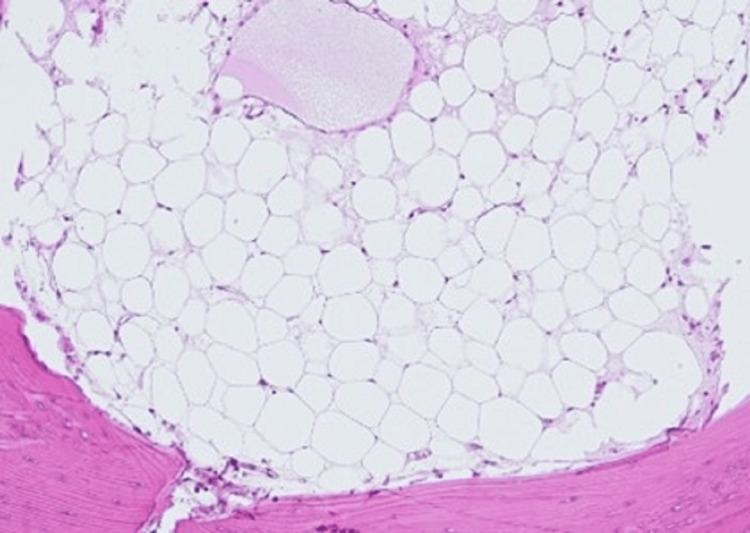
Adipose lobules adjacent to skeletal muscle fibers, suggesting peripheral soft tissue involvement.

**Figure 20:**
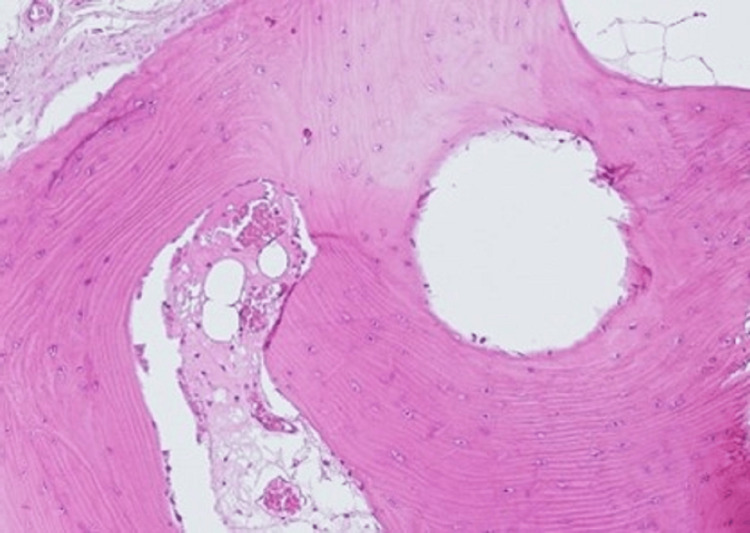
High-power view of compact bone with concentric lamellae and Haversian canals representing mature osseous differentiation.

Postoperative follow-up visit

The patient was followed up in six months’ time, where she did not have any palpable mass, and all her symptoms had resolved.

## Discussion

Osteolipoma is described as having both mature adipose tissue intermixed with lamellar bone [5]. It can originate anywhere in the body, with the most common location being in the head and anterior neck, along with the buccal mucosa [2,3,9]. In terms of proximity to the spine, we identified 10 cases in the literature; two in the lumbar spine [4,13], one in the thoracic spine [6], and seven in the cervical spine [1,7,8,10-12,14]. Table 1 shows a literature review of these articles. 

**Table 1:** Literature review of osteolipoma in the spine

Article	Author	Year	Age	Sex	Location	Size	Presentation	Treatment
Lumbar Intraspinal Osteolipoma Presenting as Cauda Equina Syndrome: A Case Report and Review of Literature	Dilip Chand Raja et al. [4]	2018	36 years old	Male	L2-3 Intraspinal epidural posterior lesion with osseous lesion attached to the right L2 inferior articular process	2 × 1.5 × 1.5 cm	Backache, lower limb claudications, left-sided unilateral numbness and foot paresthesia, deterioration in gait, and urinary incontinence following trauma. Bilateral stepping gait, bilateral weakness in L4-5, saddle paresthesia.	En bloc resection, decompression, and fusion due to facet joint removal
Spinal Ossifying Lipoma	Jaiswal et al. [13]	2005	8 years old	Female	L1 to L4 with attachment to the dorsal surface of the cord at L1, and extradural and intradural involvement at L3-4	No macroscopical information on size. However, the patient's swelling was 10 × 8 cm	Large lumbar swelling with dermal sinus, asymptomatic.	L1-L5 Laminotomy with en bloc resection
Intramedullary Osteolipoma of the Upper Thoracic Spinal Cord	Kojima et al. [6]	2016	48 years old	Female	Intramedullary tumor at T2 level	8 × 6 mm	Back pain and pain in the lateral left thigh, hyper-reflexia, and mild sensory deficit on the lateral aspect of the left thigh.	En bloc resection with the use of a microscope
Cervical Paravertebral Osteolipoma: Case Report and Literature Review	Guirro et al. [1]	2014	66 years old	Male	Right paraverterbal cervical region, posterior to C3-T1	5 × 10 cm	Painless lump for 2 years, then pain and dizziness with cervical flexion.	En bloc resection with right C3 posterior branch excision
Intraspinal Osteolipoma. Case Report	Lin et al. [7]	2001	20 years old	Female	3-cm oval extradural mass, anterior to the thecal sac at the C6-7 level	4.5 × 2.5 × 2 cm	Progressive dysesthetic numbness in both legs (R>L). gait clumsiness with frequent stumbling and falls. Dull aching pain localized in the dorsal cervical spine during neck flexion. atrophy of bilateral interosseous muscles and muscles of the lower extremities. Weakness of fingers during abduction and grasping. Both lower limbs were spastic and weak. Pinprick, position sense, and vibratory sense were also decreased bilaterally in the lower extremities. The deep tendon reflexes were hyperactive in the lower extremities. Sustained clonus and Babinski’s sign were present bilaterally.	C5-T1 laminectomy en bloc resection
Isolated Dorsal Column Dysfunction Due to an Intraspinal Osteolipoma	Aiyer et al. [8]	2016	61 years old	Male	Posterior midline lesion at the C5 level	2 × 1 × 1 cm	Neck pain, paresthesia in both upper and lower extremities, and gait clumsiness.	C4-C6 laminectomy with en bloc resection
Ossifying Lipoma of the Cervical Spine	Brones et al. [10]	2010	21 months old	Female	C2-C3	7 × 7 cm	Brought in by parents, thinking she had a communication delay. Posterior cervical neck mass with two cutaneous dimples with minimal discharge.	C2-3 laminectomy with mass resection along with its superior and inferior sinus tract, but a small portion was left behind to prevent subcutaneous depression.
Ossifying Lipoma Independent of Bone Tissue	Kameyama et al. [11]	2000	46 years old	Male	Submucosal region of the neck completely separated from the vertebrae	3 × 2 × 2	Asymptomatic neck mass	En bloc resection of the mass
Pure Intramuscular Osteolipoma	Yang et al. [12]	2013	51 years old	Female	Posterior neck muscle midline between C2-C6 spinous processes	4 × 6 × 3 cm	Painless progressive enlarging mass in the posterior neck. Neck stiffness.	En bloc resection of the mass
Cervical Paravertebral Osteolipoma: Case Report and Literature Review	Altarawneh et al. [14]	2022	60 years old	Female	Posterior to C3-C5	3 × 3.5 × 2.5 cm	Neck pain	En bloc resection of a mass from the right lamina of C3-C5

Osteolipoma typically presents as a slow-growing, painless mass. As the lesion enlarges, it may give rise to mechanical symptoms depending on its anatomical location. Reported clinical manifestations in the literature most commonly include neurological complaints such as numbness or clumsiness in the upper or lower extremities, as well as mechanical pain. In contrast, our case demonstrates two notable and unique features. First, the patient presented with intermittent periorbital numbness accompanied by dizziness - symptoms that, to our knowledge, have not been previously documented in association with osteolipoma. The underlying mechanism for these manifestations remains unclear. However, we hypothesize that the lesion’s anatomical proximity to the vertebral artery and/or the dura mater could have resulted in subtle alterations in regional blood flow or neural signal transmission, thereby contributing to the observed symptoms.

Second, intraoperative findings revealed that the mass was adherent to the dorsal dura, extending from the occiput to the C2 vertebral level. Additionally, the lesion was in close proximity to the vertebral artery - an anatomical relationship that has not been described in prior case reports. Despite this proximity, there was no evidence of compression of either the spinal cord or the vertebral artery.

Osteolipomas are typically identifiable through imaging techniques, such as CT scans or MRI, which demonstrate the characteristic fatty and calcified components [5]. However, a definitive diagnosis requires histopathological examination to confirm the presence of a mixture of adipose cells and lamellar bone or osseous elements [5].

## Conclusions

This case underscores the importance of a multidisciplinary approach in the diagnosis and management of osteolipomas. Comprehensive imaging and biopsy are critical for accurate diagnosis, while surgical excision remains the definitive treatment for symptomatic lesions. The patient's favorable outcome highlights the efficacy of this approach, although long-term follow-up is necessary to monitor for potential recurrence.

This case contributes to the limited literature on osteolipomas, providing valuable insights into their clinical presentation, diagnostic challenges, and management strategies. The review of the cases in the literature further enriches our understanding of this rare entity, emphasizing the variability in presentation and the importance of individualized treatment plans.
